# Risk-Benefit Assessment of Carotid Revascularization

**DOI:** 10.5935/abc.20180208

**Published:** 2018-10

**Authors:** Pedro Piccaro de Oliveira, José Luiz da Costa Vieira, Raphael Boesche Guimarães, Eduardo Dytz Almeida, Simone Louise Savaris, Vera Lucia Portal

**Affiliations:** Instituto de Cardiologia - Fundação Universitária de Cardiologia (IC/FUC), Porto Alegre, RS - Brazil

**Keywords:** Carotid Artery Diseases, Atherosclerosis, Endarterectomy, Carotid, Stroke, Indicators of Morbidity and Mortality, Risk Assessment

## Abstract

Severe carotid atherosclerotic disease is responsible for 14% of all strokes, which result in a high rate of morbidity and mortality. In recent years, advances in clinical treatment of cardiovascular diseases have resulted in a significant decrease in mortality due to these causes.

To review the main studies on carotid revascularization, evaluating the relationship between risks and benefits of this procedure.

The data reviewed show that, for a net benefit, carotid intervention should only be performed in cases of a periprocedural risk of less than 6% in symptomatic patients. The medical therapy significantly reduced the revascularization net benefit ratio for stroke prevention in asymptomatic patients. Real life registries indicate that carotid stenting is associated with a greater periprocedural risk. The operator annual procedure volume and patient age has an important influence in the rate of stroke and death after carotid stenting. Symptomatic patients have a higher incidence of death and stroke after the procedure. Revascularization has the greatest benefit in the first weeks of the event.

There is a discrepancy in the scientific literature about carotid revascularization and/or clinical treatment, both in primary and secondary prevention of patients with carotid artery injury. The identification of patients who will really benefit is a dynamic process subject to constant review.

## Introduction

Carotid endarterectomy was introduced in 1954 for stroke prevention, but it wasn`t until the 90`s that the first randomized clinical trials (RCTs) evaluated its effectiveness. The first published RCTs on the subject were NASCET (1991), VACS (1991) and ECST (1993), all of which demonstrated benefit of surgical intervention in secondary prevention setting.^1^^-^^3^ Regarding primary prevention, a small RCT was published in 1993^4^ followed by two larger ones (ACAS, 1995; ACST, 2004)^5^^,^^6^ that demonstrated a greater benefit of surgical intervention when compared to optimal medical treatment.

Several studies comparing carotid angioplasty and stenting (CAS) and carotid endarterectomy (CEA) were published in the 2000's, leading to a recommendation for routine use of embolic protection devices. Five clinical trials (SAPPHIRE,^7^ EVA-3S,^8^ SPACE,^9^ CREST^10^ and ACT I^11^) found that percutaneous intervention is an alternative to surgical intervention in both symptomatic and asymptomatic patients. On the other hand, the ICSS trial found a higher risk of stroke and death after CAS in symptomatic patients.^12^ Paraskevas et al.^13^ compiled data from several “real-world” registries in a systematic review and found that percutaneous procedures resulted in higher rates stroke and death when compared do CEA, albeit with conflicting results from each registry.^13^

While many studies have focused on comparing the two modalities of intervention, the definition of optimal medical treatment (OMT) has evolved and currently reduces relative risk of stroke related to extracranial atherosclerosis by up to 70%.^1^^,^^2^^,^^10^^,^^14^

Ascertaining risk-benefit ratio between CAS and CEA is challenging. There are thirty-four international guidelines on the subject, with significant variability regarding choice of carotid revascularization procedure.^15^ This review aims to provide an updated risk-benefit assessment across the different treatment options (CEA, CAS and OMT) for symptomatic and asymptomatic carotid stenosis.

## Methods

This article was based on a literature review carried out through an online search of the main articles and guidelines published in the last 30 years, aiming to evaluate the relationship between risk and benefit of carotid revascularization. Due to the differences in the indexing processes in the bibliographic databases, we opted for the search for free terms, without the use of controlled vocabulary (descriptors).

## Results

Stroke is the third cause of death in the Western world and the leading cause of permanent neurological disability.^16^ About 85% of strokes are ischemic in origin and 80% of non-hemorrhagic strokes affect brain areas irrigated by carotid arteries. Most strokes are due to thromboembolism of atherosclerotic lesions in internal carotid arteries. Usually, these occur in smaller carotid plaques with lower than 50% stenosis, considered non-surgical stenosis. The remaining cases are considered stenotic plaques that should be evaluated for surgical treatment.^14^

### Evolution of optimal medical treatment

Pivotal studies on the incidence of stroke in patients with severe symptomatic carotid stenosis, without carotid revascularization, were published in the beginning of the 1990's.^1^^-^^3^ At that time, acetyl salicylic acid was the cornerstone of OMT. In NASCET study, two year stroke incidence was 26% in OMT group, compared to 9% in CEA group.^1^ In 1995, primary prevention study ACAS^5^ found a much lower (17.5%) five-year stroke incidence in its OMT group. In 2004, ACST^7^ reported a further drop of stroke risk to 11.8% (2.4% annually), and by the time 10-year results were reported, in 2010,^17^ there was an even greater reduction in OMT group (7.2% in the last five years of follow-up). ACST also showed that in those cases of stroke with untreated severe ipsilateral carotid stenosis, OMT reduced stroke risk by almost 70%, resulting in an annual stroke incidence of 0.7% in the last five years of follow-up^17^ (Table 1).

**Table 1:** Evolution of Clinical Treatment^23^^-^^24^

Trial	Publication Year	Annual incidence of stroke in the clinically treated group
ACAS^5^	1995	3,5%
ACST first 5 years^6^	2004	2,4%
ACST last 5 years^17^	2010	1,4%

Stroke risk reduction was followed by a large reduction in myocardial infarction incidence during the same period, which is largely attributable to improvement of OMT and risk factor control.^18^

A reduction of almost 30% in mortality from atherosclerotic coronary artery disease was reported in Brazil between 1990 and 2009.^19^ Between 2003 to 2013, mortality rates due to coronary heart disease fell by 38% and the actual number of deaths decreased by 22.9% in the United States.^18^

Studies with angiotensin-converting enzyme inhibitors (ACE inhibitors) have proved the benefit of this class of drugs on ventricular remodeling, showing also a reduction of 20% in cardiovascular events.^20^^,^^21^ A meta-analysis of more than 30,000 patients demonstrated a protective effect of ACE inhibitors against ischemic events, even in patients without ventricular dysfunction.^22^ Currently, several guidelines acknowledge the role of these drugs in preventing cardiovascular disease.^23^^-^^25^

Nevertheless, routine use of statins is considered the greatest landmark in OMT. A meta-analysis of 26 RCTs (over 170,000 subjects), published in 2010, demonstrated the efficacy and safety of statins, as well as the correlation between the dose used and the protective effect.^26^ Two randomized clinical trials reported in 2016 reinforced these findings. The *Effect of Statin Treatment on Modifying Plaque Composition* (STABLE) study tested high-dose rosuvastatin through a follow-up with intravascular imaging. Besides stabilizing the atherosclerotic plaque, rosuvastatin could also induce some reversal of the atherosclerotic process.^27^ A second study, *Cholesterol Lowering in Intermediate-Risk Persons without Cardiovascular Disease* (HOPE 3), demonstrated that routine use of statin in primary prevention subjects with intermediate risk of cardiovascular diseases resulted in 24% reduction in outcomes, including stroke.^28^

### Risks and benefits of intervention

Several international societies indicate carotid intervention in symptomatic patients, ipsilateral stroke or TIA within the previous 6 months, presenting at least 50% extracranial carotid stenosis.^15^ Considering the great advances in clinical treatment in the last decades, the most important guidelines postulate that the intervention should only be performed when the periprocedural risks are smaller than 6%.^15^^,^^29^^-^^31^ (Table 2)

**Table 2:** Management of patients with Symptomatic extracranial carotid stenosis^23^^-^^24^

Carotid Stenosis	Recommendations (Class and Evidence Level)*	Periprocedural Risk to maintain clinical benefit
< 50%	OMT (IA)	
50-59%	CEA + OMT (IIaB)	< 6%
	CAS + OMT (IIbB)	
60-69%	CEA + OMT (IIaB)	< 6%
	CAS + OMT (IIbB)	
70-99%	CEA + OMT (IA)	< 6%
	CAS + OMT (IIaB)	
Occlusion	OMT (IA)	

OMT: Optimized medical therapy; CEA: Carotid endarterectomy, CAS: Carotid angioplasty and stenting. (Classes of Recommendation: I - The benefit is greater than the risk and the treatment/procedure should be performed or administered; IIa - The benefit is greater than the risk, but further studies are needed, so that it reasonable to perform procedure or administer treatment; IIb - the benefit is equal to or greater than the risk and treatment/procedure may be considered. Levels of Evidence: A - Data derived from multiple randomized clinical trials or meta-analyses; B - Data derived from a single randomized clinical trial or multiple non-randomized studies.)

* For all patients: When procedure is indicated, CAS should only be performed if there is a high risk for CEA.

In cases of severe asymptomatic carotid stenosis, the joint guideline of the American Heart Association and American Stroke Association for primary prevention of stroke, published in 2014,^30^ and the guideline of the European Society of Cardiology, published in 2017,^31^ recommend that the periprocedural risk should be less than 3% for a net benefit in the revascularization process. (Table 3)

**Table 3:** Management of patients with Asymptomatic extracranial carotid stenosis^23^^-^^24^

Carotid Stenosis	Recommendations (Class and Evidence Level)*	Periprocedural Risk to maintain clinical benefit
< 60%	OMT (IA)	
60-69%	OMT (IA);	< 3%
	CEA + OMT (IIaB) ou CAS + OMT (IIbB)	
70-99%	OMT (IA)	< 3%
	CEA + OMT (IIaB) ou CAS + OMT (IIbB)	
Occlusion	OMT (IA)	

OMT: Optimized medical therapy; CEA: Carotid endarterectomy, CAS: Carotid angioplasty and stenting. (Classes of Recommendation: I - The benefit is greater than the risk and the treatment/procedure should be performed or administered; IIa - The benefit is greater than the risk, but further studies are needed, so that it reasonable to perform procedure or administer treatment; IIb - the benefit is equal to or greater than the risk and treatment/procedure may be considered. Levels of Evidence: A - Data derived from multiple randomized clinical trials or meta-analyses; B - Data derived from a single randomized clinical trial or multiple non‑randomized studies.)

* For all patients: When procedure is indicated, CAS should only be performed if there is a high risk for CEA.

The risks associated with carotid intervention are heterogeneous, which makes it necessary to separate the patients into subgroups. (Table 4) The first important criterion in the definition of these subgroups is the presence or absence of symptoms, defined by the occurrence of a stroke or a transient ischemic attack (TIA) within the previous six months, affecting the territory supplied by the affected carotid artery.^1^ The second criterion is based on the definition of high-risk patients for carotid endarterectomy: congestive heart failure, ischemic cardiopathy, the need for associated cardiac surgery, severe pulmonary disease, contralateral carotid artery occlusion, paralysis of recurrent laryngeal nerve, carotid restenosis after procedure, cervical radiotherapy, prior cervical surgeries or age greater than 80 years.^32^

**Table 4:** Risk Subgroups for Carotid Intervention

Subgroup	Definition
Symptomatic	Occurrence of a stroke or a transient ischemic attack (TIA)within the previous six months, affecting the territory supplied by the affected carotid artery
High-risk for Carotid Endarterectomy	Congestive heart failure, ischemic cardiopathy, the need for associated cardiac surgery, severe pulmonary disease, contralateral carotid artery occlusion, paralysis of recurrent laryngeal nerve, carotid restenosis after procedure, cervical radiotherapy, prior cervical surgeries or age greater than 80 years

**Figure 1:**
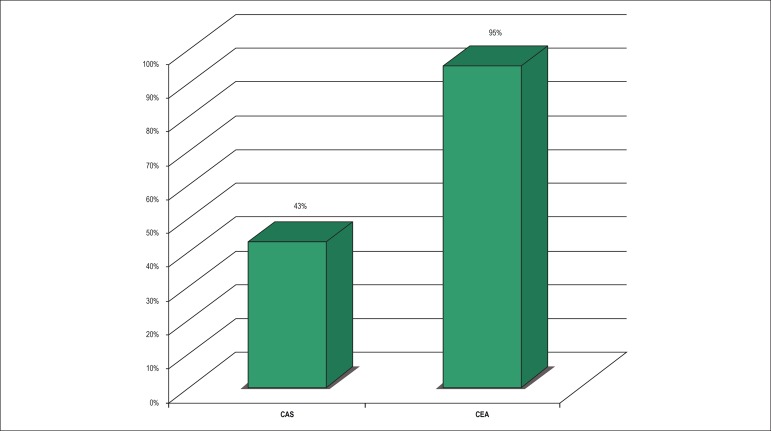
Percentage of Registries with a Lower than 3% Incidence of Stroke and Death in 30 days after Asymptomatic Carotid Intervention. CAS: Cardiotid angioplasty and stenting; CEA: Carotid endarterectomy. Paraskevas KI, Kalmykov EL, Naylor AR. Stroke/Death Rates Following Carotid Artery Stenting and Carotid Endarterectomy in Contemporary Administrative Dataset Registries: ASystematic Review. Eur J Vasc Endovasc Surg. 2015;51(1):3-12.

**Figure 2:**
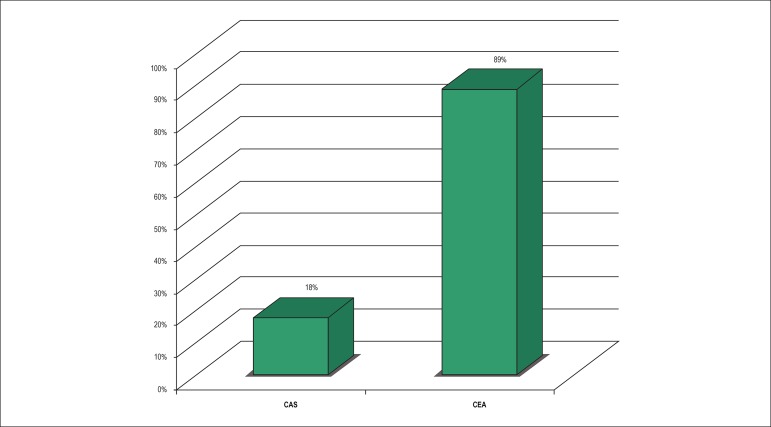
Percentage of Registries with a Lower than 6% Incidence of Stroke and Death in 30 days after Symptomatic Carotid Intervention. CAS: Cardiotid angioplasty and stenting; CEA: Carotid endarterectomy. Paraskevas KI, Kalmykov EL, Naylor AR. Stroke/Death Rates Following Carotid Artery Stenting and Carotid Endarterectomy in Contemporary Administrative Dataset Registries: ASystematic Review. Eur J Vasc Endovasc Surg. 2015;51(1):3-12.

A systematic review published in 2015 examined the rates of stroke and death after CAS and CEA in twenty-one international records, which together represent more than 1,500,000 procedures performed between 2008 and 2015.^13^ In asymptomatic patients not at high risk for endarterectomy, carotid stenting had a periprocedural risk lower than 3% in 43% of the cases, and a risk greater than 5% in 14% of the registries. For surgical revascularization in the same group, 95% of the registries reported risks lower than 3%. (Figure 1) In the group of symptomatic patients not at high risk, 72% of the registries after carotid angioplasty showed a greater than 6% incidence of stroke and death in 30 days. On the other hand, only 11% of the registries showed a risk above 6% among the patients submitted to endarterectomy. (Figure 2) Only three of the twenty-one registries analyzed reported data regarding patients with high risk for carotid endarterectomy. In one of them, the rate of events was greater than 3% in asymptomatic patients, for both CAS and CEA. In the group of symptomatic patients, all registries reported rates of stroke and death greater than 6% after CAS and two records showed rates above 6% after carotid endarterectomy.

### Carotid stenting: the age and operator effect

The elderly population usually presents vessel tortuosity and a large burden of atherosclerosis, characteristics that increase complications after angioplasty procedures. Age has been associated with periprocedural stroke and death after CAS, this same finding was not reported after CEA.^33^ A Cochrane meta-analysis of 16 randomized clinical trials^34^ and a subanalysis of the CREST trial^35^ described an association of age ≥ 70 years and increased periprocedural risk after CAS. A meta-analysis of four randomized trials (EVA-3S, SPACE, ICSS and CREST) found that the periprocedural risk of stroke or death after CAS were 3% for patients younger than 60 years and 12% for those older than 70 years, whereas the periprocedural stroke and death risk remained stable at 5% across the entire age spectrum in the CEA group.^33^

The possibility that the operator is a crucial factor for the good result of the carotid percutaneous intervention was taken into account in the design of the protocols of clinical trials involving CAS. In an attempt to standardize the group of operators, the EVA-3S study^8^ included only interventionists with a minimum of 12 carotid angioplasties performed previously. The SPACE study^9^ required a minimum of twenty and five previous procedures. Although most studies report the total volume of procedures performed by the operator, the few ones that specifically addressed this point were not able to show an association between the operator's prior experience and lower rates of complications.^36^^-^^38^

The combined analysis of three large randomized trials (EVA-3S, SPACE and ICSS), published in 2012,^39^ showed great differences in the incidence of death or stroke when the operators were stratified by annual volume of procedures. Procedures performed by operators with at least six carotid angioplasties per year had an incidence of stroke and death in 30 days of 5.1%, while the procedures performed by those with three or less, showed a 10.1% incidence. It is important to observe that all operators included in the analysis had already performed a minimum number of procedures, i.e., had already surpassed the learning curve. Unlike the annual volume, the total volume of carotid procedures performed during the life of the operator had no association with an increase of complications such as stroke and death, in concordance with other previously published studies.^40^

### Symptomatic patients revascularization - a time sensitive benefit

The results of the main studies with symptomatic patients demonstrate that the greatest benefit of intervention occurs in the first weeks after the index event.^41^^-^^43^ After the first 14 days, there is a rapid decrease in the benefit of the intervention, and more than 70% of the protective effect is seen within the first 30 days; after two years, the symptomatic patient presents the same risk level as the asymptomatic patient.^41^^-^^43^ However, this recommendation has been poorly implemented with less than 20% undergoing revascularization within two weeks the onset of the stroke or TIA.^44^ A Danish nationwide initiative was able to increase the percentage of CEA within the recommended timeframe from 13% in 2007 to 47% in 2010.^45^ The evidence of the early procedure safeness is more robust for CEA than for CAS which has conflicting results in different studies.^46^^-^^48^

Secondary prevention is indicated in cases of transient ischemic accident or small strokes, due to the high risk of intracranial hemorrhage when performing carotid intervention in the first few weeks after a major ischemic stroke and to the questionable clinical benefit in the long term.^49^

### Patient with asymptomatic severe carotid lesion

The ACAS study, published in 1995,^5^ showed that the adjusted risk of stroke and death associated with the intervention was 2.3%, with the endarterectomy preventing 59 cerebral vascular accidents in five years for every 1,000 procedures performed. Despite the very low risk as compared to that observed in practice and to those of the old pharmacological practices, 94% of the CEA were unnecessary. With an adjustment of the periprocedural risk to 0%, eighty-two cerebral vascular accidents would be prevented for every thousand endarterectomies, but still 92% of the patients would be submitted to a procedure without benefits. The same principle can be applied to the 10-year results of the ACST which showed that, with a reduction of the periprocedural risk to 0%, 74 cerebral vascular accidents would be prevented for every thousand endarterectomies, meaning that 93% of the procedures would have been unnecessary.^17^

The large clinical trials currently conducted have been limited to the comparison between carotid angioplasty and surgery. The lack of a clinical therapy group in the ACT I study, published in 2016, was strongly criticized.^50^ The new editions of the studies SPACE, SPACE-2 (ISRCTN78592017), CREST and CREST-2 (NCT02089217) planned the inclusion of a third group in clinical therapy, but the SPACE-2 study was suspended by a low rate of inclusions. Presently, the CREST-2 trial has included more than 780 of the 2,480 patients referred.

The current guidelines of the European Society of Cardiology for asymptomatic patients with severe lesions and a moderate surgical risk recommend endarterectomy (Class IIa) in the presence of clinical characteristics and/or imaging results suggestive of an increased risk of late ipsilateral stroke. Angioplasty should be considered (Class IIa) for patients with high risk for endarterectomy, provided that the rates of periprocedural death or stroke are < 3% and the patient's life expectancy is greater than five years, for any one of the groups.^31^

The population with severe asymptomatic carotid stenosis is not homogeneous. Some lines of research try to identify patients with higher risk through more detailed imaging studies to locate markers of vulnerable plaques and microembolization.^51^^,^^52^ That would allow a more cost-effective carotid revascularization in patients currently classified as asymptomatic.

## Discussion

The present review focuses on the primary and secondary prevention of ischemic stroke through carotid revascularization, which could impact 14% of all cerebral vascular accidents.^16^

The first studies on this subject were published in the beginning of the 1990's. From the year 2000, studies have focused on the comparison between angioplasty and carotid endarterectomy, without the inclusion of a clinical therapy group for comparison. In this period, there has been significant improvement of clinical treatment and better control of risk factors. The use of acetylsalicylic acid for cardiovascular prevention was already routine decades before a decline in rates of cardiovascular events was observed, suggesting that other classes of drugs are responsible for this change. In the last decades, several studies have shown the impact of statins on cardiovascular outcomes, with a reduction in incidence of up to 50%.^26^

The data reviewed in the present study show that, for a net benefit of the procedure, carotid intervention should only be performed in cases of a periprocedural risk of less than 6% in symptomatic patients or 3% in asymptomatic patients. A systematic review published in 2015 showed that carotid revascularization is more efficient in symptomatic patients but is associated to a higher incidence of death and stroke. In addition, the results did not show a trend to improved outcomes after carotid stenting between 2008 and 2015, suggesting that this modality of intervention, although less invasive, has higher rates of complications even in patients with high surgical risk.^13^

The data concerning the effect of operator in CAS show that prior experience is important and can influence the rate of serious complications. A difference of almost 100% in the incidence of 30-day stroke and death outcomes between different groups of operators has already been observed in clinical trials.^40^ The annual volume of carotid procedures performed by the operator is the factor that best correlated with lower rates of complications.^40^

The indication for carotid intervention in symptomatic patients showed a greater benefit in the first weeks of the event. In this context, the joint guideline of the American Heart Association and American Stroke Association for prevention of stroke in symptomatic patients, published in 2014, recommends as class IIa that carotid revascularization occurs within two weeks of the index event, if there are no complications that contraindicate the procedure.^30^ The 2017 guideline of the European Society of Cardiology (*ESC Guidelines on the Diagnosis and Treatment of Peripheral Arterial diseases, in collaboration with the European Society for Vascular Surgery*), maintained this recommendation.^31^

The indication for carotid intervention is still questionable in the case of asymptomatic patients, since the studies published up to now have shown a high rate of unnecessary procedures.^53^ Currently, some studies try to identify asymptomatic patients with higher risk who could undergo a more cost-effective carotid revascularization procedure.

## Conclusion

Severe lesion of the extracranial carotid artery is responsible for 14% of all cerebral vascular accidents. Carotid revascularization has been performed for over 50 years, and several studies have proven that the intervention is capable of preventing this outcome, but with a not inconsiderable risk of serious complications. More recently, carotid angioplasty procedures have broadened the range of invasive options, but the expected reduction in periprocedural risk was not observed. Additionally, the increased incidence of atherosclerosis resulted in a great heterogeneity of patients who are possible candidates for endarterectomy or stenting, and the evolution of pharmacological therapy changed the risk-benefit ratio of intervention in many cases of atherosclerotic disease. Concerning patients treated with the current best medical therapy, carotid intervention should only be performed when it is documented a periprocedural risk of less than 6% in symptomatic patients. Although major guidelines endorse intervention in asymptomatic patients provided that the periprocedural risk is less than 3%, the narrow magnitude of the absolute stroke prevention places carotid intervention as a questionable procedure in an unselected asymptomatic population.
